# Epidermolysis Bullosa, Dental and Anesthetic Management: A Case Report

**Published:** 2014-09

**Authors:** Katayoun Esfahanizade, Ali Reza Mahdavi, Ghassem Ansari, Masoud Fallahinejad Ghajari, Abdolreza Esfahanizadeh

**Affiliations:** a Dept. of Pediatric Dentistry, Islamic Azad University, Dental Branch, Tehran, Iran.; b Mofid’s Children Hospital, Shahid Beheshti University of Medical Sciences, Tehran, Iran.; c Dept. of Pediatric Dentistry, School of Dentistry, Shahid Beheshti University of Medical Sciences, Tehran, Iran.; d Instructor of Child Neurology and Neurodevelopmental Disabilities. UMDNJ/ Robert Wood Johnson Medical School, NJ, USA.

**Keywords:** Epidermolysis bullosa, Dystrophic, Dental management, Anesthetic management

## Abstract

Epidermolysis bullosa (EB) is a group of rare inherited skin and mucous membrane disorders in which blister formation may arise spontaneously or following a minor friction. Various patterns of inheritance are explicated for the disease. The disease has a profound effect on oral mucosa and may result in high prevalence of dental caries. General anesthesia is sometimes the only choice for dental treatments in patients with EB. The following case report describes the dental and anesthetic management of an 12.5 -year-old girl with dystrophic type of EB. The patient was followed up every 6 months. New carious lesions were detected one year after the treatment, on the last visit. Presenting a perfect dental care to children with this disorder can be challenging for the in charge specialist, both pediatric dentist and anesthesiologist.

## Introduction

Epidermolysis Bullosa (EB) consists of a group of uncommon skin-related diseases. These diseases are acquired or genetically transmitted in autosomal dominant and recessive traits [1]. First reported by Hebra in 1871 [2]; they are characterized by severe destruction of skin and mucous membranes. The vesiculobullous lesions may appear following a trauma, exposure to heat or even for no obvious reason. The pathophysiology of EB depends on the contributing defect in the epithelial or sub-epithelial connective tissue, showing varying degrees of severity from blister formation to premature death [2-3].

Earlier EB was classified into three different types of EB: Simplex (EBS) or epidermolytic, junctional EB (JEB) or lamina lucidolytic and dystrophic EB (DEB) or dermolytic [1]. Recently a fourth group is included which is the mixed level of cleavage (Kindler syndrome). With advancement in diagnostic technology, 30 different subtypes of this disease have also been proposed [3].

The dystrophic type has two subtypes. The autosomal dominant subtype is fairly benign [4]. The recessive subtype is the worst shape of EB in which minor friction brings out blisters and severs skin ulcerations. The disease is manifested at birth and the lesions heal slowly and are accompanied by scar formation [2]. Acquired syndactyly in hands and feet following cicatricial tissue formation may eventually lead to loss of fingers and toenails and also recurrent skin infection [2, 4-5]. A profound susceptibility to dental caries is common because of the presence of a basic defect in cementum and inability to tolerate the trauma of brushing. Subsequently, the oral hygiene is usually neglected in these patients [22, 4, 6]. Several investigators reported hypodontia, defective crowns, enamel hypoplasia and severe caries in the involved individuals [6]. The scare formation and the tissue shrinkage seriously involve the oral, pharyngeal and esophageal mucosa. Oral examination is difficult due to the continuous blister formation, healings and scar formation resulting in marked restriction of mouth opening and contracture of the lips and cheeks [5-6]. Ankyloglossia may also be present in the involved individuals.

The incidence of EB varies between 1:50,000 to 1:500,000 of live births and would implicate all racial and ethnic groups. The EB is often manifestsat birth or during the first year of life [5].

## Case Report

A 12.5 year-old girl was reported to Mofid children’s Hospital, Shahid Beheshti University of Medical Sciences, Tehran, Iran, with the chief complaint of pain and swelling in her left upper back teeth region. The patient was born with normal delivery with birth weight of 2.4 kilogram and was the only child of the family. Her parents were identified to be first-degree cousins. The patient was referred to the dermatologist because of the presence of sustained formation of bullae and blisters with scarring. The diagnosis of recessive subtype of dystrophic epidermolysis bullosa was made three hours after birth. No similar history was depicted in her family. 

On physical examination, the patient was weighing 17 Kg and with the height of 110cm portrayed the growth rate below the third percentile.

**Figure 1:**
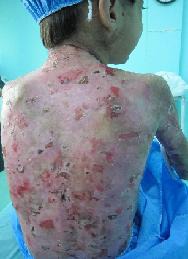
View of patient’s back demonstrating many old and new lesions

On routine medical examination, patient appeared normal. She had numerous blistering and multiple disfiguring scars and bullae on her body (Figure 1).

**Figure 2:**
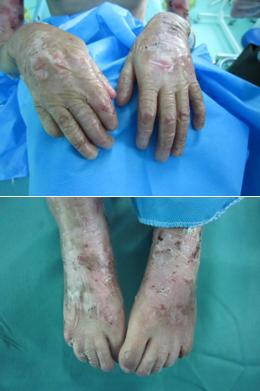
Patient’s hands and feet show that the finger and toe nails are lost and various lesions on the skin are present.

The skin over the hands and feet was highly atrophic and the fingernails and toenails were absent (Figure 2).

**Figure 3:**
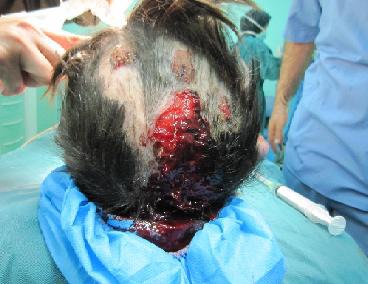
Rupture of few bullae during preparing of the child for general anesthesia

The hair was relatively thin and the fresh lesions could be seen on the scalp. Few bullae ruptured during the preparation of the patient for general anesthesia (Figure 3).

The range of the movement of extremities was normal. Psychologically, the patient appeared to have a normal cognitive function and she was attending the school. The laboratory examination of the blood, at the time of admission, disclosed that the patient had anemia (Hb=5.7 gm./dl, HCT= 27.8%) despite undertaking iron therapy, therefore, She needed blood transfusion. The packed red blood cells (150 cc) was given 6 days before surgery to raise the Hb level to an acceptable level. 

A succeeding complete blood count (CBC) showed the Hb and hematocrit levels were raised to 11.5 gm/dl and 39.8% respectively. The total white blood cells and differential count were normal. The difficulty in obtaining blood samples from this patient disallowed extensive hematologic evaluation. Medications such as iron and zinc supplements, vitamin A-D cream and silver sulfadiazine ointment for the skin lesions was prescribed for daily usage.

Dental history included restorative treatment of upper incisors, fissure-sealant therapy of all first permanent molars and composite filling of the primary canines two years ago, performed under GA. The patient exhibited no complications following the treatment and was discharged from the hospital in the same day.

**Figure 4:**
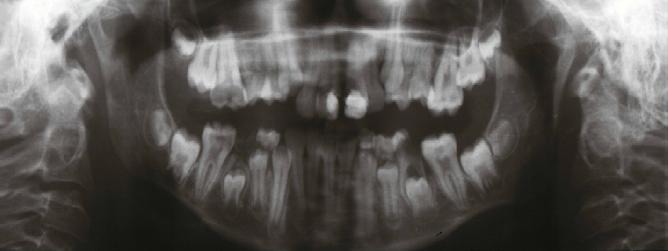
Panoramic radiograph of the patient’s dentition, demonstrating delayed dental development and deep carious lesions on left upper and lower permanent first molars and lower right second primary molar.

Even though the patient was cooperative, the oral examination and taking radiographs were challenging because of the limited mouth opening. The radiological panoramic view showed normal number of developing teeth with little delay in eruption time of permanent teeth. Deep caries on upper and lower left first permanent molars and lower right second primary molar were also detectable (Figure 4).

There was a dentoalveolar abscess in relation to the upper first permanent molar. Severe crowding was evident in both upper and lower anterior region of the jaws with plaque accumulation. The tongue movement was severely limited because of the ankyloglossia. The oral hygiene was not practiced regularly since the patient deterred to brush to avoid bullae formation. The premedication consisted cephalexin IV (500mg) four times a day.

**Dental treatments and anesthetic management**

An IV catheter was employed on patient’s forehand. No adhesive tape was allowed to contact the skin. The only possible way of monitoring was pulse oximetry, using the patient’s ear lobe and capnography. The naso-tracheal intubation was achieved when the patient’s face, the laryngoscope blade, the induction mask, and the endotracheal tube were all generously lubricated with a 1% hydrocortisone cream. A number 4, non- cuffed tube was utilized which was softened in a warm saline for few minutes prior to placement. An oropharyngeal pack was then engaged to seal of oropharyngeal area.

The anesthetic medications used for induction were: Fentanyl ampule 15µg (Fentanyl citrate; Janssen Pharmaceutical, N.Y), Sodium Thiopental 6mg/kg (Ltd. India), Atracorium 7mg (Atracural; Alborz Darou, Tehran, Iran). The patient was maintained with Isoflurane 0.8% (AErrane; Baxter MFD, Puerto Rico). The dental elevators and forceps were used with utmost care to avoid any undesirable trauma. The absorbable sutures were used and tied loosely.

The operative procedure was carried out in routine way, nevertheless with extreme caution to avoid any tissue damage. No mouth probe was possible to be used. After the completion of treatment and before extubation, the airway was checked for presence of any bullae by the anesthesiologist. The patient was easily awakened and responded to verbal stimuli immediately. The lips exhibited some swelling and there were erosive lesions around the lips and over the tongue with some vesicles that burst and left tissue debris. Upper and lower left first permanent molars and lower right second primary molar were extracted. The whole procedure took 30 minutes. The sterile gauze was packed on the extraction site until the clot was formed. The patient was under supervision for post-operative care in the first 24 hours to be monitored for any airway obstruction. During this period, the patient received intra venous Cephalexin (50 mg/kg) in divided doses every 12 hours. Her vital signs were checked and they were stable. 24 hours after the surgical treatment the child complained of severe pain in the mouth and was unable to eat solid foods. She was able to drink milk and eat ice cream only. New blisters were formed on her tongue and in various areas of the oral mucosa. Palliative treatment and using milk of magnesia was prescribed. The symptoms lasted for 10 days during which a 0.05% NaF mouth rinse (Oral-B; Procter & Gamble Company, US) was recommended. The child was instructed to drink more fluids with her daily food.

**Figure 5:**
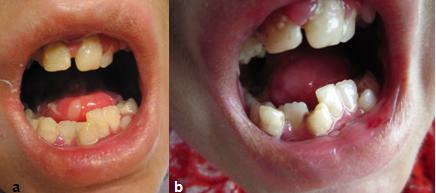
a: First follow-up visit of the patient. Note a slight gingival inflammation and new lesions on the tongue  b: Second follow-up visit of the patient. Note a severe gingival inflammation and new carious lesions on the anterior teeth plus dislodgement of previous fillings.

The child was followed up every 6 months for one year. New and recurrent dental caries appeared during this period. On each visit; new restorations were performed in the clinic without local anesthesia. On the final follow up visit, silver-reinforced glass ionomer base cement (Riva Silver; SDI limited, Australia) was placed under composite fillings in the anterior teeth. It was also used as a permanent filling for lower right first permanent molar. The remaining primary molars were mobile and close to be exfoliated. The fluoride varnish (DuraSheild; 5%Naf, Sultan Healthcare, Hackensack, NJ, USA) was applied on each visit (Figure 5).

## Discussion

The case presented here illustrated many features of the recessive type of dystrophic EB [7-9]. Extensive dental caries and abscess formation, often brings these patients to the dentist’s attention which is directly related to their incapability of having a decent oral hygiene [10, 4].

Wright et al. showed that caries susceptibility was much higher in EB [11] which could be due to the severity of soft tissue involvement, consumption of soft diet rich in sucrose, necessary to provide sufficient energy intake and their difficulty in mechanical elimination of dental plaque due to their incapability in holding the toothbrush [5]. A comprehensive oral care instruction and a preventive program should be given to these patients as soon as possible. To enhance the oral clearance of food debris, an increased fluid intake during meals could be helpful [12]. Water-jet systems, soft toothbrushes [5] and fluoride mouth rinses are recommended and all may help control caries. Topical application of neutral sodium fluoride and fluoride varnishes is well tolerated by these patients [12]. Chlorhexidine (CHX) mouth rinses can also help control caries [5, 12]. To reduce the pain and number of blisters, Sucralfate (the treatment choice for active duodenal ulcers) has been suggested as an effective prophylactic and therapeutic agent [1].

A delayed dental maturity of 2 1/4 months, based on Dermirjian's system, was reported in the involved individuals, which was also discerned in our case [12].

The oral findings of the patient was consistent with many features defined for this disease in previous studies [12-14]. These patients manifest vesicles that tear quickly, leaving erosive surfaces on the mucosa of lips and oral cavity, vestibular obliteration, denudation of the tongue, microstomia and ankyloglossia are other common manifestations of the disease [5, 12-14].

Reviewing the literature on proposed dental treatments for this disease, tooth extraction is often suggested because of the presence of extensive caries, oral bullae and enamel hypoplasia [15]. The most important recommendation is to take an extreme care to minimize the trauma to the oral cavity. During the dental treatment in outpatient setting, administration of local anesthesia may also cause blister formation; therefore should be avoided whenever possible [13]. Injecting local anesthesia solution should also be slow and deep into the tissues to prevent any mechanical tissue trauma [5].

Ankyloglossia, severe oral and perioral scarring, limited mouth opening and obliteration of the oral vestibule would complicate the dental treatments. Oral ulceration during dental manipulation is inevitable; however, it could be prevented by lubrication of mucosa with hydrocortisone cream, triamcinolone or petroleum jelly before starting any procedure. 

Secondary infections can be prevented by employing oral antiseptics or application of topical antibiotics on the existing bullae [16, 5]. Small-sized instruments including burs and handpiece are highly recommended. The dental restorations in these patients should be highly polished [5]. Some authors believe that intubation may predispose the patient to high risk of pharyngeal and tracheal lesions [17]. However, if the procedure is performed gently by using a smaller lubricated laryngoscope, the rate of new bullae formation would be reduced significantly [18-19, 5]. Outpatient settings are more preferred because the risks of hospital infection would then be decreased significantly. Nonetheless, multiple extractions in an out-patient setting might be very traumatic. If general anesthesia is the selected modality, Prabhu et al. [12] have listed a few recommendations in their article as follows. Face masks should be prepared with few layers of Vaseline gauze. A minimal chin lift and head tilt should be exerted and gentle manipulation of the head with a hand below the occiput and the jaw should be considered. Tapes should not be used on skin surfaces in any circumstances. Electro cardiograph (ECG) monitoring may be omitted in cases of severe skin involvement. For pulse oximetry, an adult clip-on probe can be used if total pseudosyndactyly of fingers and toes is not present. Another alternative would be placement of an adhesive strip over a clear plastic bag, covering the hand or foot.

Nasotracheal intubation is the modality of choice for most of the patients with EB due to limited mouth opening. Moreover, the nasal mucosa would be less vulnerable to bulla formation and oral rehabilitation procedures would be more easily accomplished, when using this modality.

## Conclusion

Epidermolysis bullosa depicts a group of rare genetic disorders that involves the skin and mucous membrane by vesicle and bullae formation. The oral and dental manifestations of EB disrupt the patient's oral health and hygiene and exhibit a challenge for the dental professionals. Administration of general anesthesia for patients with EB requires distinctive attention and inclusive cares.

## References

[1] Marini I, Vecchiet F (2001). Sucralfate: a help during oral management in patients with epidermolysis bullosa. J Periodontol.

[2] Crawford EG, Burkes EJ Jr, Briggaman RA (1976). Hereditary epidermolysis bullosa: oral manifestations and dental therapy. Oral Surg Oral Med Oral Pathol.

[3] Rao R, Mellerio J, Bhogal BS, Groves R (2012). Immunofluorescence antigen mapping for hereditary epidermolysis bullosa. Indian J Dermatol Venereol Leprol.

[4] Album MM, Gaisin A, Lee KW, Buck BE, Sharrar WG, Gill FM (1977). Epidermolysis bullosa dystrophica polydysplastica. A case of anesthetic management in oral surgery. Oral Surg Oral Med Oral Pathol.

[5] Torres CP, Gomes Silva JM, Mellara TS, Carvalho LP, Borsatto MC (2011). Dental care management in a child with recessive dystrophic epidermolysis bullosa. Braz Dent J.

[6] Howden EF, Oldenburg TR (1972). Epidermolysis bullosa dystrophica: report of two cases. J Am Dent Assoc.

[7] Kaslick RS, Brustein HC (1961). Epidermolysis bullosa. Review of the literature and report of a case. Oral Surg Oral Med Oral Pathol.

[8] Horn HM, Tidman MJ (2002). The clinical spectrum of dystrophic epidermolysis bullosa. Br J Dermatol.

[9] Lindemeyer R, Wadenya R, Maxwell L (2009). Dental and anaesthetic management of children with dystrophic epidermolysis bullosa. Int J Paediatr Dent.

[10] Hitchin AD (1973). The defects of cementum in epidermolysis bullosa dystrophica. Br Dent J.

[11] Wright JT, Fine JD, Johnson L (1993). Hereditary epidermolysis bullosa: oral manifestations and dental management. Pediatr Dent.

[12] Prabhu VR, Rekka P, Swathi S, Ramesh, Swathi S (2011). Dental and anesthetic management of a child with epidermolysis bullosa. J Indian Soc Pedod Prev Dent.

[13] Kostara A, Roberts GJ, Gelbier M (2000). Dental maturity in children with dystrophic epidermolysis bullosa. Pediatr Dent.

[14] Serrano Martínez MC, Bagán JV, Silvestre FJ, Viguer MT (2003). Oral lesions in recessive dystrophic epidermolysis bullosa. Oral Dis.

[15] Wright JT (1984). Epidermolysis bullosa: dental and anesthetic management of two cases. Oral Surg Oral Med Oral Pathol.

[16] Wright JT, Gantt DG (1983). Epidermolysis bullosa. Associated with Enamel Hypoplasia and Taurodontism. J Oral Pathology & Medicine.

[17] Herod J, Denyer J, Goldman A, Howard R (2002). Epidermolysis bullosa in children: pathophysiology, anaesthesia and pain management. Paediatr Anaesth.

[18] Iohom G, Lyons B (2001). Anaesthesia for children with epidermolysis bullosa: a review of 20 years' experience. Eur J Anaesthesiol.

[19] Stavropoulos F, Abramowicz S (2008). Management of the oral surgery patient diagnosed with epidermolysis bullosa: report of 3 cases and review of the literature. J Oral Maxillofac Surg.

